# Policy measures and instruments used in European countries to increase biosimilar uptake: a systematic review

**DOI:** 10.3389/fpubh.2024.1263472

**Published:** 2024-02-28

**Authors:** Sara Machado, Agostinho Cruz, Pedro Lopes Ferreira, Carminda Morais, Rui Esteves Pimenta

**Affiliations:** ^1^ESS, Polytechnic of Porto, Porto, Portugal; ^2^LAQV-REQUIMTE, ESS, Polytechnic of Porto, Porto, Portugal; ^3^Faculty of Economics, University of Coimbra, Coimbra, Portugal; ^4^Centre for Health Studies and Research of the University of Coimbra/Centre for Innovative Biomedicine and Biotechnology (CEISUC/CIBB), Coimbra, Portugal; ^5^School of Health, Polytechnic of Viana do Castelo, Viana do Castelo, Portugal

**Keywords:** biological products, biosimilar pharmaceuticals, drug substitution, health policy, economics

## Abstract

**Introduction:**

While biosimilar medicines can contribute to the sustainability of healthcare systems, their utilization rate varies across European countries. This study aims to identify and systematize policy measures and instruments used in European countries to increase biosimilar market share.

**Methods:**

A systematic review was conducted according to PRISMA 2020 recommendations. Medline-PubMed, Web of Science and ScienceDirect databases were searched using inclusion criteria that required full articles published in English between January 2006 and November 2023. Reviews, letters, reports, editorials and comments or opinion articles were excluded from this study.

**Results:**

Of the 1,137 articles, only 13 met the eligibility criteria for analysis, which covered a total of 28 European countries. Pricing regulation measures were found in 27 of these countries with tendering, price-linkage and internal reference price being the most used. Tendering was used by 27 countries to procure biosimilars in inpatient setting. Prescribing guidelines and recommendations were the widely used instrument. Some European countries adopted physician incentives, quotas, and prescription by international non-proprietary name.

**Conclusion:**

Automatic substitution was not commonly recommended or applied. Interchangeability and switching will become increasingly relevant issues. It is important that the positive results from some countries serve as an example for the future of these medicines in the European market.

**Systematic review registration:**

https://inplasy.com/, Identifier INPLASY2023120032.

## 1 Introduction

The demographic changes in the European population, as well as an increase in life expectancy combined with low birth rates, represent one of the important economic and social challenges of our time (1). Despite the increase in life expectancy, many years in old age are lived with chronic illnesses causing disabling effect (2). In fact, as populations age, the prevalence of chronic diseases increases, requiring health systems to promote high-quality management (3). In healthcare, one measure is the use of cost-effective drug therapies, as drugs play a fundamental role in mitigating the burden of disease (4, 5).

Biological medicines (BMs) constitute a significant component of drug expenditures in Europe, representing 35% of drug spending based on the list price (6). These medicines are experiencing above-average annual growth compared to other drug groups (6). Moreover, their use in chronic diseases treatment highlights the importance of these products for modern societies, making them an indispensable part of today’s medical arsenal, with a rapidly accelerating market growth in the last decade (6, 7).

The development of BMs requires considerable investment by the pharmaceutical industry, which is why they are protected by exclusivity rights (7). The average clinical development cost for a BM ranges from US$800 million (around €680 million). In contrast, biosimilars have a development cost ranging from US$100 million to US$200 million (around €85–170 million), and a similar development duration of 8–10 years (8). Biosimilar medicines are produced after the patent of the reference BMs has expired and are authorized for the European market through a centralized procedure conducted by the European Medicines Agency (EMA) (7).

Europe has been a pioneer in the adoption of biosimilars, with a well-established market and strong regulation. The market for biosimilars began in 2006 and since then, the European continent has had over 15 years of experience in the development and production of high-quality, safe, and effective biosimilars (9). This positive experience in the acute and chronic treatment of millions of European patients enables addressing one of the main health challenges in Europe, which is ensuring equitable access (9).

As of May 2023, 75 biosimilars have been approved and authorized for 19 active substances, spanning various therapeutic areas such as diabetes, inflammatory bowel diseases (IBD), psoriasis, rheumatology, ophthalmology, hematology, and oncology. These biosimilars encompass a range of biological products, including insulins, epoetin, granulocyte-colony stimulating factor, heparins, anti-tumor necrosis factor, and monoclonal antibodies (10). Over the next years, between 2023 and 2028, there will be an increase in the number of BMs losing their protection, with approximately 14 biologic molecules already having competitors in development. This number is higher than the amount observed in the previous 5 years period (6).

The cumulative savings at list prices resulting from the impact of biosimilars competition in Europe were estimated at over €30 billion as of 2022 (6). Biosimilars contribute to the sustainability of healthcare systems, offering an attractive strategy to reduce costs and increase accessibility for patients, which can improve health outcomes. Furthermore, the introduction of biosimilars in the market not only increases competition but also encourages innovation, potentially stimulating advancements in the formulation and development of next-generation BMs (6, 11, 12).

However, it is important to implement measures to improve understanding and increase the use of biosimilars by healthcare systems, as has been done with generic drugs (11, 13). Currently, the literature indicates variations in the utilization rates of biosimilars, both among and within European countries (6, 14–18), which may be influenced by different policies, medical perspectives, competition among suppliers and prices (19, 20).

The EMA has not yet regulated issues such as interchangeability, switching and automatic substitution. However, it has in 2022 expressed support for interchangeability (7, 21). Therefore, each European country implements its own measures and policies, which are evaluated for their ability to control costs and ensure access to medicines (22).

Policy measures and instruments on both the supply and demand sides have an impact on the market share of biosimilars (23–25). Price and reimbursement policy measures, stakeholder incentives for biosimilars use, as well as the level of education and awareness, lead to variations in the use of these drugs (24). Therefore, it is important to systematize information related to this topic to ensure the future of biosimilars in European healthcare systems and their efficiency. The main objective of this systematic review was to identify and systematize the policy measures adopted in European countries related to the increase of biosimilars market share and the instruments used in this process.

## 2 Essential concepts

In the statement issued by the EMA on interchangeability, experts from the European Union (EU) consider that it is not necessary to conduct systematic switch studies to support prescriber-level interchangeability (21). However, each member state is free to allow or not allow pharmacy-level substitution. Therefore, it is crucial to define these practices clearly, due to their impact on the use of these medicines. In the following, these practices are described in essential concepts, along with an explanation of policy measures and instruments classified on both the supply and demand sides.

The interchangeability is the possibility of exchanging one drug for another, hoping to achieve the same clinical effect. This may mean replacing a reference product with a biosimilars (or vice versa) or replacing one biosimilars with another. The substitution can be done through automatic substitution or switching. Thus, automatic substitution is the practice of dispensing one medicine instead of another equivalent and interchangeable medicine at the pharmacy level, without consulting the physician. In turn, switching occurs when the prescriber decides to change one drug to another with the same therapeutic purpose in patients undergoing the same treatment (26).

### 2.1 Policy measures and instruments used to increase the biosimilar market share

#### 2.1.1 Policy measures and instruments on the supply-side

In the context of the global pharmaceutical market, national policies aimed at setting prices and reimbursements, even with specific national objectives, have implications that extend beyond the country’s borders, affecting various aspects related to drugs on a transnational level (27).

Internal reference pricing involves a comparison of a drugs price with that of a product containing the same active substance or a therapeutically similar drug within the same country. On the other hand, external reference pricing establishes prices by looking at the cost of the same drug in one or several other countries. Price linkage determines the price in relation to the cost of the reference medicine (28, 29).

Moving toward value-based pricing, this approach sets prices based on the medications value when compared to existing therapies for the same clinical indication. This evaluation is typically conducted through Health Technology Assessment (HTA) (28, 29).

Tendering and negotiation are mechanisms that set prices through competition among suppliers. Furthermore, reimbursement structures play an important role, impacting the price of the medication by specifying the amount that will be reimbursed by the healthcare system (28, 29).

#### 2.1.2 Policy measures and instruments on the demand-side

Incentives for physicians to prescribe biosimilars can be implemented through prescription quotas, establishing a targeted level for the quantity of prescriptions. These quotas are often accompanied by financial incentives or penalties if not achieved. Another approach involves prescribing guidelines and recommendations, where prescribers are required to adhere to guidelines, often set forth by national authorities. International Non-proprietary Names (INN) prescribing mandates physicians to prescribe drugs by their international non-proprietary names, i.e., active substance instead of commercial name (29, 30).

Gain share agreements operate as models where an increase in biosimilar usage leads to gains that are reinvested in healthcare for the benefit of all involved parties. Additionally, educational programs play a crucial role, offering informative sessions aimed at both patients and healthcare professionals (29, 30).

## 3 Methods

### 3.1 Screening and study selection

The study was conducted on February 6, 2023, on Medline-PubMed, Web of Science (Web of Science Core Collection), and ScienceDirect databases. To update the results obtained, a new search was later conducted on December 2, 2023, using the same databases. After the initial search, duplicate articles were removed. The screening of the obtained articles was conducted by title and abstract by two independent researchers (SM and AC). The search strategy used was follows: (“drug substitution/standards” [MeSH Terms] OR “drug substitution/methods” [MeSH Terms] OR “drug substitution/economics” [MeSH Terms] OR “Drug Substitution” [MeSH Terms]).

AND

(“biosimilar pharmaceuticals/therapeutic use*” [MeSH Terms] OR “biosimilar pharmaceuticals/standards” [MeSH Terms] OR “biosimilar pharmaceuticals/economics*” [MeSH Terms] OR “biosimilar pharmaceuticals/administration and dosage*” [MeSH Terms]).

The search strategy was adapted for each database.

### 3.2 Selection criteria

To select studies, inclusion and exclusion criteria were defined. Therefore, the inclusion criteria encompassed full-text articles in English, published between January 2006 and November 2023. This timeframe was chosen as the first biosimilar was approved in Europe in 2006. Additionally, the articles had to reference at least one European country, not limited to EU members. Only studies describing policy measures and/or instruments aimed at increasing the biosimilars market share and used in the country were considered. Exclusion criteria included reports, investigative letters, opinion or comment articles, editorials, systematic reviews, reviews, qualitative studies, incomplete articles, as well as studies referring to countries outside Europe. Two researchers (SM and AC) independently assessed titles and abstracts to exclude non-relevant articles based on eligibility criteria. The remaining articles underwent full-text screening by the same two researchers, when significant discrepancies exist, third-party arbitration was required (RP).

### 3.3 Quality assessment

The quality of the included studies was assessed using the Joanna Briggs Institute (JBI) Checklist for cross-sectional studies (31). For each study, the risk of bias was assessed separately by two researchers (SM and CM).

### 3.4 Data analysis

For the included articles, a data extraction form was created. One researcher filled out the form, and validation was conducted by the entire team. The form included key study details such as authors, publication year, covered European countries, objectives, participants (for real-world evidence studies), and results (measures and/or instruments for increasing biosimilars market share).

This systematic review was conducted in accordance with the Preferred Reporting Items for Systematic Reviews and Meta-Analyses 2020 (PRISMA) recommendations. This methodology consists of a set of evidence-based guidelines for reporting studies in systematic reviews and meta-analyses, comprising 27 items on a checklist that should be included in the report of a systematic review or meta-analysis (29). Thus, the research question was defined using the PICO strategy (Patients-P, Intervention- I, Comparison-C, Outcomes-O). The investigated population was European patients undergoing treatment with biological medicines (P), and the intervention was the economic policy measures or/and instruments (I). Comparison was the absence of economic policy measures and instruments for different European countries (C), with the aim of increasing the biosimilars market share (O). Formulating the research question: “What policy measures and instruments are used in European countries to increase the biosimilar market share?”

## 4 Results

### 4.1 Screening

**Figure 1:**
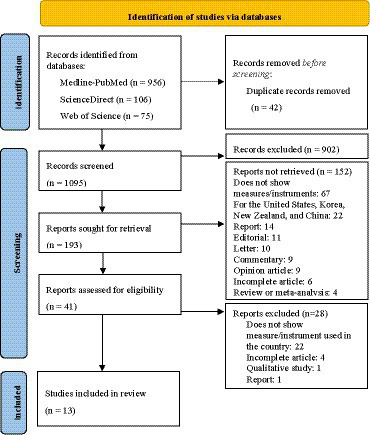
PRISMA flow diagram.

A total of 1,137 articles were retrieved from Medline-PubMed, Web of Science and, ScienceDirect databases, of which 42 were duplicates (Figure 1). After removing the duplicates,1095 articles were screened for relevance based on their titles and abstracts, resulting in the exclusion of 902 articles due to reasons related to their titles (mainly because they were reviews, clinical trials, or addressing countries that did not belong to Europe) and 152 due to reasons related to their abstracts. Of the remaining 41 full-text articles, 28 were excluded for different reasons based on eligibility criteria. Finally, 13 studies were included in the final analysis. The entire process is illustrated in Figure 1 and the final list of articles is presented in Supplementary Tables S1, S2.

### 4.2 Quality analysis

The quality of the included studies was assessed using the Joanna Briggs Institute (JBI) Checklist for cross-sectional studies (31). For each study, the risk of bias was assessed separately by two researchers. The studies overall demonstrated good quality. Nevertheless, certain criteria were labeled as “unclear,” and in other cases, they were marked as “no” when suitable statistical methods were not employed, an instance observed in two cases. The results as presented in Supplementary Table S3.

### 4.3 Studies characteristics

The found studies covered 28 European countries and focus on supply-side and demand-side policy measures. Three of these took a general approach and included multiple European countries, providing a global view (32–34). Only one of the studies simultaneously addressed two countries (35), while the remaining nine studies referred to a specific country, indicating one or several policy measures and instruments used in the country to increase the biosimilars market share (36–44). Five out of the nine studies comprised real world evidence investigations, presented a specific policy measure or instrument used in interventions in their respective countries (37–41).

Using the included articles, two tables were created containing the main features, policy measures and instruments found, as presented in Supplementary Tables S1, S2. The real world evidence studies were separated and are presented in the Supplementary Table S2. The summary of these findings is provided in Table 1 which presents the policy measures and instruments identified in European Countries.

**Table 1:** Policy measures and instruments identified in European countries.

Policy Measures and Instruments		Countries	References
Supply-side	Price regulation policies	AT, BE, BG, CZ, DE, EE, FI, FR, HR, HU, IE, IS, IT, LAT, LT, MLT, NL, NO, PL, PT, RO, RS, SE, SI, SK, SP, UK	Kawalec et al. (32), Moorkens et al. (33), Barszczewska et al. (35), Moorkens et al. (44)
	Price-linkage	AT, BE, BG, CZ, EE, FI, FR, HR, HU, IE, IT, LAT, LT, NO, PL, PT, RO, SK, SP	Kawalec et al. (32), Vogler et al. (34)
	Internal reference pricing	AT, BG, CZ, DE, EE, FR, HR, HU, LAT, LT, NL, PL, RO, RS, SI, SK, SP	Kawalec et al. (32), Moorkens et al. (33)
	External reference pricing	BG, CZ, IS, IT, HR, LAT, MLT, PT, RS, SI	Moorkens et al. (33)
	HTA evaluation	BG, CZ, EE, HR, HU, LAT, PL, RO, SK	Kawalec et al. (32)
	Reference price system	CZ, DE, DK, NL, NO, SK, SP	Vogler et al. (34)
	Reimbursement policies	FR, HU, PL, SE	Barszczewska et al. (35), Harsányi et al. (36), Moorkens et al. (44)
	Free pricing by the company	DE, UK	Moorkens et al. (33)
	Free pricing without exceeding the price of the reference product	NO	Moorkens et al. (33)
	Price is same as the price of the reference product	NL	Moorkens et al. (33)
	Price set through HTA	SE	Moorkens et al. (33)
	Tendering in inpatient setting	AT, BE, BG, CZ, DE, DK, EE, FI, FR, HR, HU, IE, IS, IT, LAT, LT, MLT, NL, NO, PL, PT, RS, SE, SI, SK, SP, UK	Kawalec et al. (32), Moorkens et al. (33), Vogler et al. (34), Jahnsen and Jørgensen (39)
	Tendering in outpatient setting	BG, CZ, DE, DK, HU, MLT, NL, PL, RS, SK	Kawalec et al. (32), Moorkens et al. (33), Vogler et al. (34)
Demand-side	Physician incentives	AT, BE, CZ, DE, EE, FI, FR, IE, IS, IT, NL, NO, UK, PT, SE, SK, SP, UK	Moorkens et al. (33), Vogler et al. (34), Moorkens et al. (44)
	Prescribing guidelines and recommendations	AT, BE, CZ, DE, DK, FI, FR, HU, IE, IT, NL, NO, PT, SE, SK, SP, UK	Vogler et al. (34), Harsányi et al. (36), Moorkens et al. (44)
	National guidelines and recommendations	DK, NO	Glintborg et al. (37, 38), Jahnsen and Jørgensen (39)
	INN prescription	BE, CZ, DE, FI, FR, IE, IT, NL, NO, PT, SK, SP, UK	Vogler et al. (34)
	Interchangeability	BG, CZ, EE, HR, HU, LAT, PL, RO, SK	Kawalec et al. (32)
	Therapeutic substitution	BG, CZ, EE, HR, HU, RO, SK	Kawalec et al. (32)
	Automatic substitution	CZ, EE, FR^a^, LAT, PL	Moorkens et al. (33), Vogler et al. (34)
	Prescription target or quota	DE, SE	Birkner and Blankart (42), Moorkens et al. (44)
	Educational programs	NL, NO, PT	Moorkens et al. (33)
	Gain share agreement	IE, SE, UK	Plevris et al. (40), Razanskaite et al. (41), Duggan et al. (43), Moorkens et al. (44)
	Financial incentives or penalties	DE, SE	Birkner and Blankart (42), Moorkens et al. (44)
	Financial incentives to dispense	FR	Vogler et al. (34)

AT, Austria; BE, Belgium; BG, Bulgaria; CZ, Czechia; DE, Germany; DK, Denmark; EE, Estonia; FI, Finland; FR, France; HR, Croatia; HU, Hungary; IE, Ireland; IS, Iceland; IT, Italy; LAT, Latvia; LT, Lithuania; MLT, Malta; NL, Netherlands; NO, Norway; PL, Poland; PT, Portugal; RO, Romania; RS, Serbia; SE, Sweden; SI, Slovenia; SK, Slovakia; SP, Spain; UK, United Kingdom.

HTA, Health technology assessment; INN, International Non-proprietary Name.

a was abolished in the 2020.

### 4.4 Location

The included articles addressed measures and instruments used in 28 European countries. Austria, Belgium, Bulgaria, Croatia, Czechia, Denmark, Estonia, Finland, France, Germany, Hungary, Iceland, Ireland, Italy, Latvia, Lithuania, Malta, Netherlands, Norway, Poland, Portugal, Romania, Serbia, Slovakia, Slovenia, Spain, Sweden, and the United Kingdom were investigated in 13 different studies (32–44).

## 5 Discussion

### 5.1 Supply-side policy measures and instruments

Several supply-side policy measures have been adopted by European countries, and 27 countries have national pricing regulations for biosimilars. Thus, most European countries regulate prices through a set of policy measures applied to different settings of the market, such as the outpatient or inpatient setting. These measures include tendering, where prices are set through competition between suppliers. For medical products used in the inpatient setting (hospitals), tendering occurs in 27 countries, but it can be done nationally or by hospital. In Sweden, tendering is done at the county level (44). In Norway, since 2007, an annual tender system for biologics, including biosimilars, has been established, and the pharmaceutical company offers the product price for a period of 12 months. This measure can be an important factor in procurement at lower prices. In addition, the Norwegian government has allocated 20 million NOK (around €2 million) to study whether the switch from the reference biologic to the biosimilars *infliximab* was safe, in 2014. This study, called NOR-SWITCH, was a randomized controlled trial that demonstrated that the biosimilars of *infliximab* was not inferior to the reference biologic in terms of efficacy, tolerability, safety and immunogenicity in patients who have been stable for at least 6 months (39, 45). This study, initiated 1 year after the approval of the biosimilars by EMA, allowed for an improvement in national treatment options for patients with IBD, increasing the use of biosimilars. Additionally, it was an important international contribution that allowed other countries to adopt the switch, increasing their biosimilars market share.

Another policy measure used by European countries to regulate prices is the internal reference price, which was identified as a measure used by most European countries. Internal reference pricing enables the consistent setting of prices for medicines with similar or identical therapeutic effects (28). The implementation of this policy measure can, therefore, contribute to price reduction, particularly if the prices of biosimilars are lower than their reference BM. In some countries, the reference BMs and the biosimilars were typically placed in the same homogeneous group (32). The study by Moorkens et al. (33), states that not only a single pricing mechanism is used but rather a combination to determine the price of biosimilars in the outpatient setting. Internal reference pricing was applied in 13 of the 23 European countries under investigation.

In turn, the price-linkage measure, which is the reduction of the price of biosimilars compared to the reference BM, is also widely adopted by European countries to establish the price of biosimilars. In France, price-linkage is used to set prices in the outpatient setting. The first biosimilar entering the market must have a price lower than 40% of the reference BM, and there is an additional 20% reduction in the price of the reference biologic. Further price reductions are applied after 18 and 24 months (34). In Hungary, the initial biosimilar introduced to the market must provide a 30% price reduction compared to the price of the reference BM, while the second and third biosimilars need to offer an additional 10% reduction based on the price of the first or second biosimilar. Any subsequent biosimilar is required to enter the market with a price lower than the least expensive reimbursed product (32). In Finland and Czechia, the first biosimilar entering the market is required to present a price 30% lower than the reference BM (34). Based on our findings, this policy measure is used for price setting in 19 European countries. However, in Germany, Denmark, the Netherlands, Sweden, and the United Kingdom, this policy measure is not applied (32, 34). In a way, the implementation of this policy measure can bring economic benefits to healthcare systems and subsequently increase the uptake of these medicines. However, it is essential to adopt a combination of measures that encompass a variety of policy goals (25). In addition to price regulation, there are also policies for the reimbursement of biosimilars in the studied countries, often accompanied by HTA.

### 5.2 Demand-side policy measures and instruments

Demand-side policy measures play a crucial role in increasing the market share of biosimilars, with prescribing guidelines and recommendations being important instruments in this process. Switching is recommended for use in Denmark, France, Finland, Norway, and the Netherlands, according to prescribing guidelines and recommendations. These are used by most European countries studied. In Hungary, prescribing guidelines were pointed out as an instrument for the biosimilars *infliximab*, particularly the recommendation to use the lower-priced biosimilars in patients starting treatment. As guidelines and recommendations can occur at the national level. In Norway, the Norwegian Health Authorities recommended that all IBD patients start treatment with the biosimilars *infliximab* (39). Similarly, in Denmark, the Danish Health Council recommended the prescription of biosimilars for naïve patients and the switch to and between biosimilars. A national guideline was used to make the switch mandatory to biosimilars for all patients with IBD (37, 38). In April 2016, all patients with inflammatory arthritis treated with the reference biological *etanercept* were mandatory switched to the biosimilars *etanercept*, for economic reasons. Eligible patients for the switch had their data in the national registry DANBIO. This national database can be a useful instrument to facilitate the implementation of switch programs for biosimilars. The policy measures found are in line with the study by Jensen et al. (46), which proposed a model for the rapid implementation of two biosimilars in Denmark, with the main measure being the non-medical switch. The study by Azuz et al. (18) made a comparison between the market share of biosimilars trastuzumab in Denmark and 17 other European countries. Three months after entering the market, its share increased to 90% in Denmark, while the Netherlands were the second country to achieve the highest share, with only 50%. It is important to note that, as in the study by Jensen et al. (46), the preparation of the implementation was identified as the main measure, involving all stakeholders and emphasizing the importance of communication between all parties involved in the switch (18, 46).

Demand-side policy measures and instruments, these largely include incentives for physicians to prescribe biosimilars. In Germany, prescription quotas and prescription targets (maximum pharmaceutical budget defined by period, region, specialty or physician) are used to encourage biosimilars prescription in the outpatient setting, accompanied by financial penalties (42). Quotas are often used by German medical associations to control costs and reduce uncertainty among regular prescribers (23). In Belgium, physicians must consider the prescription quota of “low-cost medicines.” In this sense, physicians are encouraged to prescribe at least 20% of biosimilars to naïve patients (47).

The INN prescribing measure is used in several European countries. This is one of the key policy measures that influence how physicians prescribe medicines and could lead to an increase in the biosimilars market share.

Another demand-side policy measure is gain share agreements, which constitute incentive policies. In the UK, specifically in England, this measure is adopted to finance a switch program at local level. The agreement is made between the parties involved, in this case, the University Hospital Southampton [UHS] NHS Foundation Trust and the local Clinical Commissioning Groups (CCGs) and consists of creating the necessary conditions for the implementation of the switch program. In Scotland as well, these benefit-sharing initiatives occur at the regional/local level, the agreement is established between the Tertiary IBD center in Edinburgh and the local Trust, and this measure is also used to finance a switch program. Since the switch program between the reference biologic and the biosimilars saves financial resources, this measure constitutes a way of stimulating the adoption of biosimilars. According to Moorkens et al. (17), the negotiation of gain share agreements is prompted by hospitals recognizing the insufficient resources to manage the switching program in England. Furthermore, increased investments in additional staff to facilitate the switching program have contributed to higher rates of biosimilars uptake (17). The reduction of costs is one of the main reasons behind the implementation of these incentive political measures. On the other hand, these measures decrease the autonomy of prescribing physicians and have a negative impact on patients’ rights (34).

In Sweden, local gain share agreements also take place within counties, where the cost savings generated through the switching process have been reallocated to local hospitals (44). Although used in several European countries Barcina Lacosta et al. (48) state that, in order to achieve the full potential of this measure, there needs to be greater transparency regarding the reinvestment of the resulting savings (48). It is crucial to emphasize that, much like in the study conducted by Razanskaite et al. (41), the development of the switch program involved collaboration with diverse stakeholders, including patients. Their active participation in this process ensured that the patients’ perspective was duly considered. Also, in the study by Dylst et al. (47), there was a discussion regarding patients’ perceptions of biosimilars that can impact their acceptance. Therefore, a lack of confidence in biosimilars constitutes an important barrier to the uptake of these products (47).

Although educational programs for physicians or patients may be relevant to increase the biosimilars market share, these programs were only mentioned in a study that pointed out their use in three European countries. Currently literature points for educating patients about biosimilars is essential to ensure clarity and prevent misinformation. Patients require access to clear and evidence-based information that empowers them to make informed decisions about their treatment (11, 49). Like what happened with generic drugs, it is necessary to educate the patient to have a more positive attitude toward biosimilars. Patient-focused educational programs help reduce information asymmetry, thereby increasing patient empowerment with the aim of enhancing shared decision-making.

### 5.3 Other considerations

Regarding interchangeability, it is allowed only at the physician’s discretion in most of the countries studied, according to EMA guidance (21). Interchangeability is considered a determining factor in the adoption of biosimilars in the biological drugs market (20). Moreover, automatic substitution is an important issue in this context, as in most European countries, including Portugal, pharmacists cannot perform substitution without consulting the physician. As for switching, it was found that it is generally permitted under physician supervision in most countries, although in some situations, it is mandatory and recommended by national authorities, as mentioned earlier.

This study successfully identified a range of policy measures and instruments used in the European countries under analysis. However, further research may be necessary to identify any additional policy measures and instruments that were not included. This may be because specialized sources on the subject were not used and the responsible entities in each country were not consulted. On the other hand, this review covers the period since the introduction of biosimilars in Europe in 2006, which means that there may have been subsequent changes that are not included in this work.

Finally, assessing the economic impact of policy measures and instruments on expenditures is crucial for understanding their significance. It is essential to determine whether the adopted policy measures and instruments have effectively met the intended objectives.

## 6 Conclusion

The results of this systematic review reveal that the policy measures and instruments adopted by European countries to increase the biosimilars market share vary wildly. However, there is evidence that effective policies must be adapted to the specificities of each country and, therefore, a combination of policies may be necessary to achieve success (25). Supply-side policy measures such as tendering, price-linkage and internal reference pricing are the most commonly used price regulation measures. Tendering in inpatient setting is widely used by the European countries to procure biosimilars. In outpatient setting, the most used pricing mechanism for biosimilars involve price-linkage.

Prescribing guidelines and recommendations were the most used instrument, being able indicate another measure, such as switching. Denmark is a successful example of biosimilars implementation, with an approach that includes communication among all stakeholders. To increase the biosimilars market share, many European countries have implemented demand-side policy measures that influence prescribing, including incentives for physicians, quotas and INN prescription. On the other hand, automatic substitution was found to be a policy measure that is not highly recommended or applied.

Therefore, it is recommended that the EMA provides guidance on the most effective policy measures and instruments to increase the biosimilars market share, with a focus on ensuring consistency and effectiveness across all European countries. This requires a thorough impact assessment of each measure and instrument.

It is expected that policy measures and instruments will continue to emerge from European countries, since increasing the biosimilars market share contributes to the sustainability of health systems and increases patients’ access to biological therapies. Interchangeability and switching will be increasingly relevant issues, and it is important that the positive results of some countries serve as an example for the future of these drugs in the European market.

## Data availability statement

The original contributions presented in the study are included in the article/Supplementary material, further inquiries can be directed to the corresponding author.

## Author contributions

SM: Investigation, Writing – original draft, Writing – review & editing, Methodology. AC: Formal analysis, Validation, Writing – review & editing, Conceptualization, Methodology, Project administration, Supervision. PF: Formal analysis, Validation, Writing – review & editing, Funding acquisition. CM: Formal analysis, Validation, Funding acquisition, Writing – review & editing. RP: Conceptualization, Formal analysis, Project administration, Supervision, Validation, Writing – review & editing, Funding acquisition, Resources.
