# Supplementary material for: Policy measures and instruments used in European countries to increase biosimilar uptake: a systematic review
Source: Front Public Health. 2024 Feb 28;12:1263472. doi: 10.3389/fpubh.2024.1263472 (PMC10932952; doi:10.3389/fpubh.2024.1263472)
Supplement: Supplementary file 1 [file Data_Sheet_2.ZIP › 1263472_SupMaterial.pdf]

Supplementary Material

1 Supplementary Table

Table S1. Main Features, Policy Measures and Instruments Identified in the European Countries

Author, Year	Countries	Objective	Sample	Policy Measures and Instruments
Barszczewska et al., 2020 (35)	FR and PL	Compare selected aspects of reimbursement and access to the EMA authorized biosimilar medicines in two countries – France and Poland	European countries, N=2	Price and reimbursement regulation policy - FR and PL.
Birkner & Blankart 2022 (42)	DE	Help decision-makers by analyzing the impact of introducing biosimilar prescription targets on physician prescribing behavior in the prescription of erythropoiesis-stimulating agents in Germany	European country, N=1	Physician Incentives to Prescribe Biosimilars: Financial incentives or penalties; Biosimilar prescription target/quotas - at regional level;
Duggan et al. 2021 (43)	IE	Investigate the utilisation of biosimilars following a 'best-value biological' medicine initiative for adalimumab and etanercept in the Irish healthcare setting	European countries, N=1	Physician Incentives to Prescribe Biosimilars; Gain share agreement.
Harsányi et al., 2020 (36)	HU	Evaluates the efficiency of the Hungarian biosimilar drug policy on the case of biosimilar infliximab	European country, N=1	Prescribing guidelines; Reimbursement regulation policy; The study also suggests the following measures to be implemented: When there are multiple biological medications available to treat the same therapeutic indication, it is recommended to use a financial protocol to define the order of priority; A mandatory switch should be implemented under medical supervision.

Table S1 (continued)

Author, Year	Countries	Objective	Sample	Policy Measures and Instruments
Kawalec et al., 2017 (32)	BG, CZ, EE, HR, HU, LAT, LT, PL, RO, and SK	View the requirements for the reimbursement of biosimilars and to compare the reimbursement status, market share, and reimbursement costs of biosimilars in selected Central and Eastern European countries	European countries, N=10	National price regulation policies - all countries;<br>Internal reference pricing - BG, CZ, EE, HR, HU, LT, PL, RO and SK.<br>Tendering - BG, CZ, EE, HU, LT, PL and SK (EE e LT hospitals);<br>HTA evaluation - all countries for the reimbursed biosimilars, except in LT;<br>Price-linkage for biosimilars - all countries;<br>Therapeutic substitution (a change of one substance for another substance) - allowed in all countries as physician discretion except in LAT, LT and PL;<br>Interchangeability - allowed in all countries as physician discretion except LT.
Moorkens et al., 2017 (33)	AT, BE, BG, CZ, DE, EE, FI, FR, HR, IE, IS, IT, LAT, MLT, NL, NO, PL, PT, RS, SE, SI, SP and UK	Provide an overview of different initiatives and policies that may influence the uptake of biosimilars in different European countries	European countries, N=23	National price regulation policies - all countries;<br>Internal reference pricing - AT, BG, CZ, DE, EE, FR, HR, LAT, NL, PL, RS, SI and SP;<br>External reference pricing - BG, CZ, IS, IT, HR, LAT, MLT, PT, RS and SI;<br>Free pricing by the company - DE and UK (regulated market);<br>Free pricing without exceeding the price of the reference product - NO;<br>Price is same as the price of the reference product - NL;<br>Price set through HTA - SE;<br>National tendering in outpatient setting - MLT and RS;<br>National tendering in inpatient setting - all countries;<br>Physician incentives - AT, BE, DE, EE, FI, FR, IS, NO, UK, PT (hospitals) and SP, IT and SE (regional);<br>Educational programs and publications for clinicians/patients - NL, NO and PT;<br>Automatic substitution (pharmacy level) - EE, FR (specific conditions), LAT and PL.

Table S1 (continued)

Author, Year	Countries	Objective	Sample	Policy Measures and Instruments
Moorkens et al., 2019 (44)	SE	Analyze the market dynamics of originator and biosimilar etanercept (outpatient setting) in the different counties of Sweden, and examine the influence of local policy measures and practices, in addition to national policy	European countries, N=1	National Pricing and Reimbursement policy<br>Prescribing guidelines and recommendations – at counties level<br>Gain share agreement – at counties level
Vogler et al., 2021 (34)	AT, BE, CZ, DE, DK, FI, FR, IE, IT, NL, NO, PT, SE, SK, SP and UK	Informs about different policy measures employed by European countries to design the biologicals market and explores potential savings from the increased use of biosimilar medicines in Germany	European countries, N=16	Price-linkage - AT, BE, CZ, FI, FR, IE, IT, NO (may be priced at the same price of the reference), PT, SK and SP;<br>Tendering in inpatient setting - all countries;<br>Tendering in outpatient setting - CZ, DE, DK, NL and SK;<br>Reference price system - CZ, DE, DK, NL, NO, SK and SP;<br>INN prescription - BE, CZ, DE, FI, FR, IE, IT, NL, NO, PT, SK, SP and UK;<br>Prescribing guidelines and recommendations for switching - is possible in all countries, however, with physician supervision in most of them, in DK, NL, NO and FR is recommended. In FI, obligation to prescribe the most economical therapeutical alternative for all (not only naive) patients, when biosimilars are available. Prescribing of a more expensive alternative must be justified in writing in the patient's medical record;<br>Automatic substitution (pharmacy level) - CZ (but not recommended by physicians and pharmacists) and DE (as of 2022 substitution can takes place automatically);<br>Financial incentives to dispense biosimilars (at pharmacy level) - FR;

AT=Austria; BE=Belgium; BG=Bulgaria; CZ= Czechia; DE=Germany; DK=Denmark; EE=Estonia; FI=Finland; FR=France; HR=Croatia; HU=Hungary; IE=Ireland; IS=Iceland; IT=Italy; LAT=Latvia; LT=Lithuania; MLT=Malta; NL=Netherlands; NO=Norway; PL=Poland; PT=Portugal; RO=Romania; RS=Serbia; SE=Sweden; SI=Slovenia; SK=Slovakia; SP=Spain; UK=United Kingdom.
CD=Crohn's disease; DANBIO=Danish nationwide registry includes all rheumatologic patients receiving biological drugs, including biosimilars; IA=Inflammatory arthritis; IBD=Inflammatory bowel disease; INN=International Non-proprietary Name; IRD=Inflammatory rheumatic diseases; HTA=Health technology assessment; RA=Rheumatoid arthritis.

Table S2. Main Features, Policy Measures and Instruments Identified in the Real World Evidence Studies

Author, Year	Countries	Objective	Sample	Policy Measures and Instruments
Glintborg et al., 2019 (37)	DK	Explore if switching lead to increased healthcare utilization and costs	European country, N=1<br>Patients with IA from the DANBIO registry, N=1620	National guidelines and recommendations - Non-medical mandatory switch for all patients with IA. Through the national registry DANBIO.
Glintborg et al., 2019 (38)	DK	Investigate in Etanercept-treated patients the proportions of patients who switched to biosimilars (switchers) or maintained Etanercept treatment (non-switchers)	European country, N=1<br>Patients with IA from the DANBIO registry, N=2061	National guidelines and recommendations - Non-medical mandatory switch for all patients with IA. Through the national registry DANBIO.
Jahnsen and Jørgensen, 2017 (39)	NO	Compare clinical and biochemical parameters before and after switch from originator to biosimilar infliximab in terms of efficacy, tolerability and safety	European country, N=1<br>Patients with IBD, N=68	Tendering - Annual tender system;<br>National guidelines and recommendations - Norwegian Health Authorities recommended for patients with IBD starting treatment with infliximab biosimilar.
Plevris et al., 2018 (40)	UK (Scotland)	Prospectively evaluate clinical outcomes in a cohort of patients with Crohn's disease switching from Remicade to CT-P13	European country, N=1<br>Patients with CD, N=110	Switching program financed through a gain share agreement between the involved parties.
Razanskaite et al., 2017 (41)	UK (England)	To present the outcomes of a service evaluation of switching IBD patients established on originator infliximab to biosimilar, using a managed switching program funded via a gain share agreement in a UK teaching hospital	European country, N=1<br>Patients with IBD, N=263	Switching program financed through a gain share agreement between the involved parties.

DK=Denmark; NO=Norway; UK=United Kingdom.
CD=Crohn's disease; DANBIO=Danish nationwide registry includes all rheumatologic patients receiving biological drugs, including biosimilars; IA=Inflammatory arthritis; IBD=Inflammatory bowel disease.

Table S3. Quality analysis results

Year	Authors	1. Were the criteria for inclusion in the sample clearly defined?	2. Were the subjects and the setting described in detail?	3. Was the exposure measured in a valid and reliable way?	4. Were objective, standard criteria used for measurement of the condition?	5. Were confounding factors identified?	6. Were strategies to deal with confounding factors stated?	7. Were the outcomes measured in a valid and reliable way?	8. Was appropriate statistical analysis used?	Overall appraisal (Include/Exclude)
2020	Barszczewska, O	✓	✓	✓	✓	✓	✓	✓	✗	✓
2022	Birkner, B	✓	✓	✓	✓	✓	✓	✓	✓	✓
2021	Duggan, B	✓	✓	✓	✓	✓	✓	✓	✓	✓
2019	Glintborg, B; Ibsen, R	✓	✓	✓	✓	✓	✓	✓	✓	✓
2019	Glintborg, B; Loft, A	✓	✓	✓	✓	✓	✓	✓	✓	✓
2020	Harsányi, S	✓	✓	✓	✓	✓	✓	✓	✓	✓
2017	Jahnsen, J	○	✓	✓	✓	○	○	✓	✓	✓
2017	Kawalec, P	✓	✓	✓	✓	✓	✓	✓	✓	✓
2017	Moorkens, E	✓	✓	✓	✓	✓	✓	✓	✓	✓
2019	Moorkens, E	✓	✓	✓	✓	✓	✓	✓	✓	✓
2019	Plevris, N	✓	✓	✓	✓	✓	✓	✓	✓	✓
2017	Razanskaite, V	✓	✓	✓	○	○	○	✓	✗	✓
2021	Vogler, S	✓	✓	✓	✓	✓	✓	✓	✓	✓

✓ Yes
✗ No
○ Unclear
